# The impact of the COVID-19 pandemic on the trend of prescribing long-acting injections of paliperidone and risperidone in Central Serbia

**DOI:** 10.3389/fpsyt.2023.1301835

**Published:** 2023-12-21

**Authors:** Milena Stojkovic, Marija Sekulic, Mirjana Jovanovic, Aleksandar Kocovic, Danijela Djokovic, Natasa Minic, Milan Djordjic, Stefan Joksimovic, Marija Sorak, Bojan Stojanovic, Snezana Sretenovic, Aleksandra Cvetkovic, Tamara Stojanovic, Olivera Radmanovic, Branimir Radmanovic

**Affiliations:** ^1^Department of Psychiatry, Faculty of Medical Sciences, University of Kragujevac, Kragujevac, Serbia; ^2^Department of Hygiene and Ecology, Faculty of Medical Sciences, University of Kragujevac, Kragujevac, Serbia; ^3^Department of Pharmacy, Faculty of Medical Sciences, University of Kragujevac, Kragujevac, Serbia; ^4^Department of Communication Skills, Ethics, and Psychology, Faculty of Medical Sciences, University of Kragujevac, Kragujevac, Serbia; ^5^Surgical Oncology Clinic, Institute for Oncology and Radiology of Serbia, Belgrade, Serbia; ^6^Department of Gynecology and Obstetrics, Faculty of Medical Sciences, University of Kragujevac, Kragujevac, Serbia; ^7^Department of Surgery, Faculty of Medical Sciences, University of Kragujevac, Kragujevac, Serbia; ^8^Department of Internal Medicine, Faculty of Medical Sciences, University of Kragujevac, Kragujevac, Serbia; ^9^Department of Ophthalmology, Faculty of Medical Sciences, University of Kragujevac, Kragujevac, Serbia; ^10^Department of Philology and General Education Subjects, Faculty of Philology and Arts in Kragujevac, University of Kragujevac, Kragujevac, Serbia; ^11^Internal Clinic, University Clinical Center Kragujevac, Kragujevac, Serbia

**Keywords:** COVID-19 pandemic, LAI risperidone, LAI paliperidone, mental health, psychotic disorders

## Abstract

Since the end of 2019, the global spread of COVID-19 has represented a historic event that changed our way of treating patients globally. The use of long-acting injections (LAI) antipsychotics was emphasized. Our goal was to investigate the impact of COVID-19 on the frequency of prescribing LAI and compare it with a period before. All patients (198) who started LAI-risperidone or LAI-paliperidone for the period 2017–2022, in Kragujevac, the city in Central Serbia, were considered. The frequency of prescribing LAI before and during COVID-19 and the total number of prescribed LAI per year were compared. Separately, the frequency of prescribing LAI-R and the frequency of prescribing LAI-P were compared. The significant (*p* < 0,05) increase in the use of LAI risperidone and paliperidone was in 2020 and 2021 [per year 2017(3), 2018(6), 2019(26), 2020(75), 2021(55), and 2022(33)]. The significant (*p* < 0,05) increase in monthly and quarterly preparations of LAI paliperidone was in 2020 and 2021 relative to the years before the pandemic. As the pandemic weakened, the inclusion of LAI paliperidone therapy weakened during 2022. A significant increase in usage of LAI risperidone was in 2022, and in 2020 and 2021 was as it was in the period 2017–2019. During COVID-19, especially in years when COVID-19 restriction measures were stricter, there was a significant change in the application method of antipsychotic therapy in favor of LAI. Regardless of the increase in treatment costs, patients’ interests and protection were prioritized in the treatment process.

## Introduction

In most countries, the COVID-19 pandemic started suddenly and without warning. The WHO labeled it a pandemic on March 11, 2020, after the first cases were identified in late December 2019 (1). The complexity of health systems worldwide and the challenges of obtaining accurate infection and immunity data contribute to the unexpected nature of COVID-19’s development. Most countries used lockdown as a containment method considering the pandemic’s severity. The pandemic has had far-reaching repercussions on our lives, as well as our jobs. Physical distancing of the general population and isolation of cases was proposed as the most effective measure of disease prevention (2). While the effects of lockdown and isolation have been extensively studied in individuals with mental health problems, the general population, special vulnerable populations, and health care providers, it was less investigated how they impacted treating trends (doses, formulations, regiment).

Disorders from the psychotic spectrum group are mostly chronic diseases that require long-term pharmacotherapy. The advent of antipsychotics in the middle of the 20th century enabled good control of the positive symptoms of schizophrenia, eliminating or reducing them to a tolerable level in as many as 70% of patients. Antipsychotic maintenance therapy is recommended indefinitely, even for those who have achieved remission after a first psychotic episode (3). Compliance with oral maintenance therapy is estimated to be only 40–60% 1 year after symptom reduction of an acute episode of schizophrenia. Inadequate adherence during the maintenance period in patients with psychotic alienation is associated with higher rates of relapses, and frequent hospitalizations, creating potentially enormous costs and burdens for the patient and his family, as well as worse long-term outcomes. The goal of developing long-acting injections antipsychotics was to establish better compliance, a stable drug dose in the blood, and improve the cognitive status and quality of life of these patients (4). The availability of LAI paliperidone and risperidone is quite wide. Namely, in Serbia patients can receive LAI risperidone in a two-week regimen and LAI paliperidone in a monthly and three-month application regimen at the expense of the insurance and without additional payment when it is recommended by specialist of psychiatry.

Due to the lockdown that we were all in, the treatment of many somatic and psychiatric disorders has changed. The COVID-19 pandemic presented a challenge for all health professionals who provide services to patients with schizophrenia and other psychotic spectrum disorders. To prevent relapses or exacerbation and their consequences, continuity of medical care is crucial for these patients. So, we found ourselves at the point where the need for increased care and restrictions of isolation collided. The LAI antipsychotics were emphasized during the COVID-19 pandemic. LAI ensures treatment continuity for a certain longer period (1 to 3 months without in-person visits). This is advantageous under specific situations which include no access to health care for a certain time such as lockdown in the pandemic. Our goal was to investigate the impact of the COVID-19 pandemic on the frequency of prescribing LAI and compare it with a period before the pandemic.

## Methods

### Data collection and participants

Our research involved 198 patients who started risperidone or paliperidone LAI in the period 2017–2022, in Kragujevac, city of Central Serbia. The study was conducted at the Clinic for Psychiatry, University Medical Center Kragujevac. The research was approved by the Ethics Committee of the University Clinical Center Kragujevac. Data on patients, medications, and doses were collected from the database located within the Clinic for Psychiatry, the University Clinical Center, and its long-acting injection outpatient clinic.

### Inclusion and exclusion criteria

Inclusion criteria for the study were diagnosis from the spectrum of psychotic disorders confirmed by DSM-5 for which the use of LAI Risperidone and Paliperidone is registered, stable status of illness at the time of recruitment, patients treated as outpatients, having stable maintenance doses of their LAIs. Exclusion criteria were unstable (exacerbation) of illness, hospitalized patients, patients with other psychiatric comorbidities, patients with somatic comorbidities, and the use of concomitant therapy that requires frequent visits to a psychiatrist.

### Statistical analysis

For the purposes of this study, comorbidities were not considered even though they are common in this population (5). We compared the trend (frequency) of prescribing LAI before and during the COVID-19 pandemic. Separately, the frequency of prescribing LAI of risperidone and the frequency of prescribing LAI paliperidone. Also, the total number of prescribed LAI per year was reached. Chi-Squared test was used to compare using of investigated drugs before and during pandemic. All data were analyzed using IBM SPSS software, version 21. The statistical significance threshold was set at 0.05.

## Results

Overall, 198 patients receiving LAI paliperidone and risperidone LAI were identified. According to gender, there were 67 females and 131 males. The largest number of patients were middle-aged, 30–55 years old. In terms of education, 72% had a secondary education, 15% had a university degree, and 3% had no schooling or completed elementary school. The characteristics of the sample are shown in Table 1.

**Table 1:** Sociodemographic data.

Sex	67 females
	130 males
Age	18 – 65 years
Dg	Psychotic spectrum (F20 – F29)
LAI risperidone	39 (13 females, 26 males)
LAI paliperidone (monthly)	72 (36 females, 36 males)
LAI paliperidone (quarterly)	87 (24 females, 63 males)

Dg, diagnosis.

**Figure 1:**
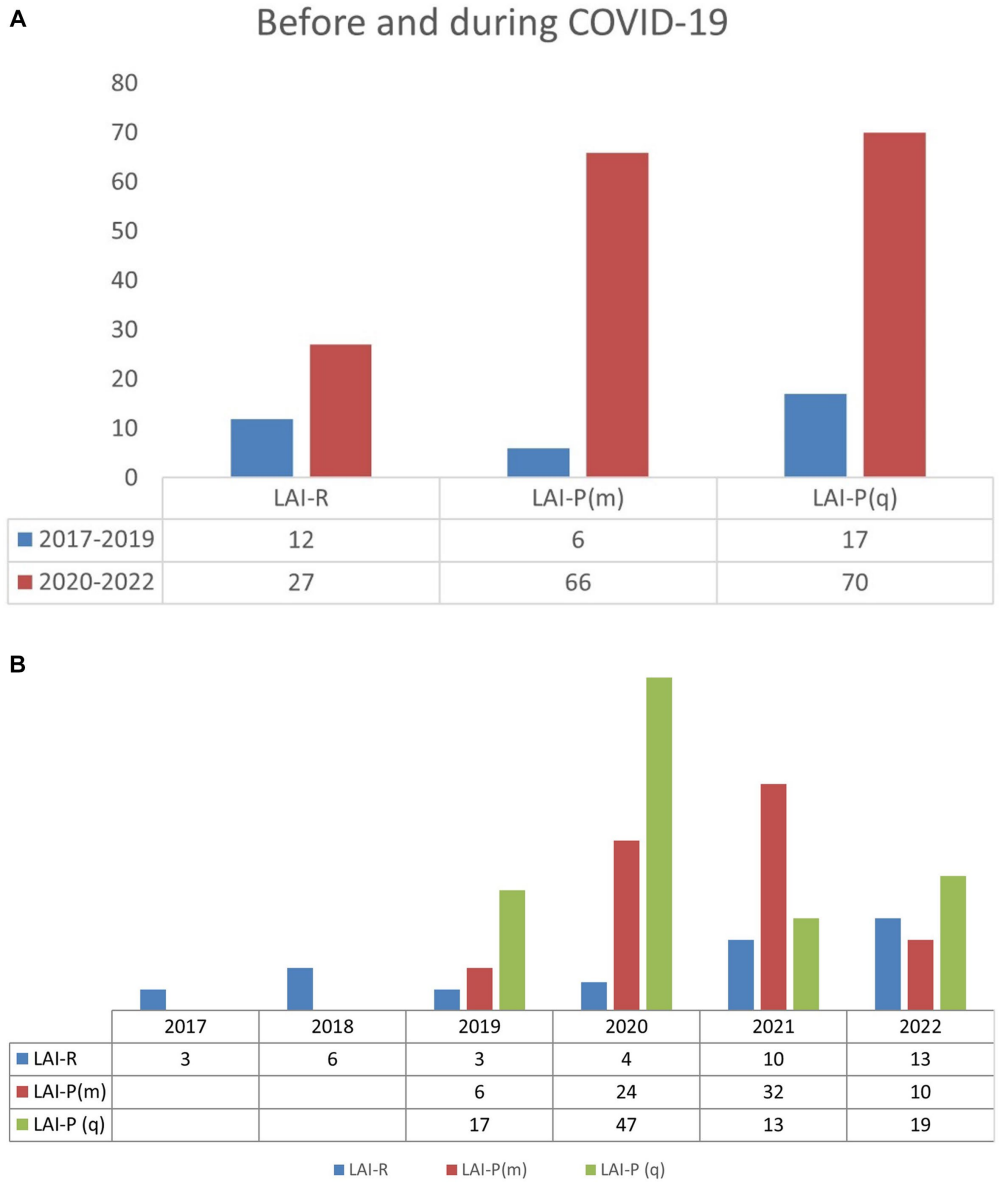
**(A)** Prescribing of LAI before and during COVID-19. **(B)** Prescribing of LAI before and during COVID-19. LAI-R, LAI risperidone; LAI-P(m), LAI paliperidone monthly; LAI-P(q), LAI paliperidone quarterly.

During the COVID-19 pandemic (period 2020–2022), there was a significant change in the application method of antipsychotic therapy in favor of LAI, compared to the period before the pandemic (from 2017 to 2019) (χ^2^ = 9,111; *p* = 0,011). The number of every followed dosing form by period of observation is presented in Figure 1A. Compared individually, monthly, and quarterly preparations of LAI paliperidone had a significant increase in usage, compared to LAI risperidone (χ^2^ = 6,215; *p* = 0,013). The number of patients that use any followed dosage form by year is presented in Figure 1B.

The total number of patients that were introduced to LAI risperidone and paliperidone therapy, according to the year of introduction and according to the long-acting drug that was prescribed is shown in Table 2. From the results, we can observe an increase in the use of LAI risperidone and paliperidone markedly in 2020 and 2021. Per year it was 2017(3), 2018(6), 2019(26), 2020(75), 2021(55), and 2022(33) patients.

**Table 2:** The number of patients and percent of patients with LAI antipsychotics therapy by year.

	2017	2018	2019	2020	2021	2022
LAI-R	3 (100)	6 (100)	3 (11.54)	4 (5.33)	10 (18.18)	13 (39.39)
LAI-P(m)	0 (0)	0 (0)	6 (23.08)	24 (32)	32 (58.18)	10 (30.3)
LAI-P(q)	0 (0)	0 (0)	17 (65.38)	47 (62.67)	13 (23.64)	10 (30.3)

Values are given in format number [percent (%) of patients in that year]. LAI-R, LAI risperidone; LAI-P(m), LAI paliperidone monthly; LAI-P(q), LAI paliperidone quarterly.

**Figure 2:**
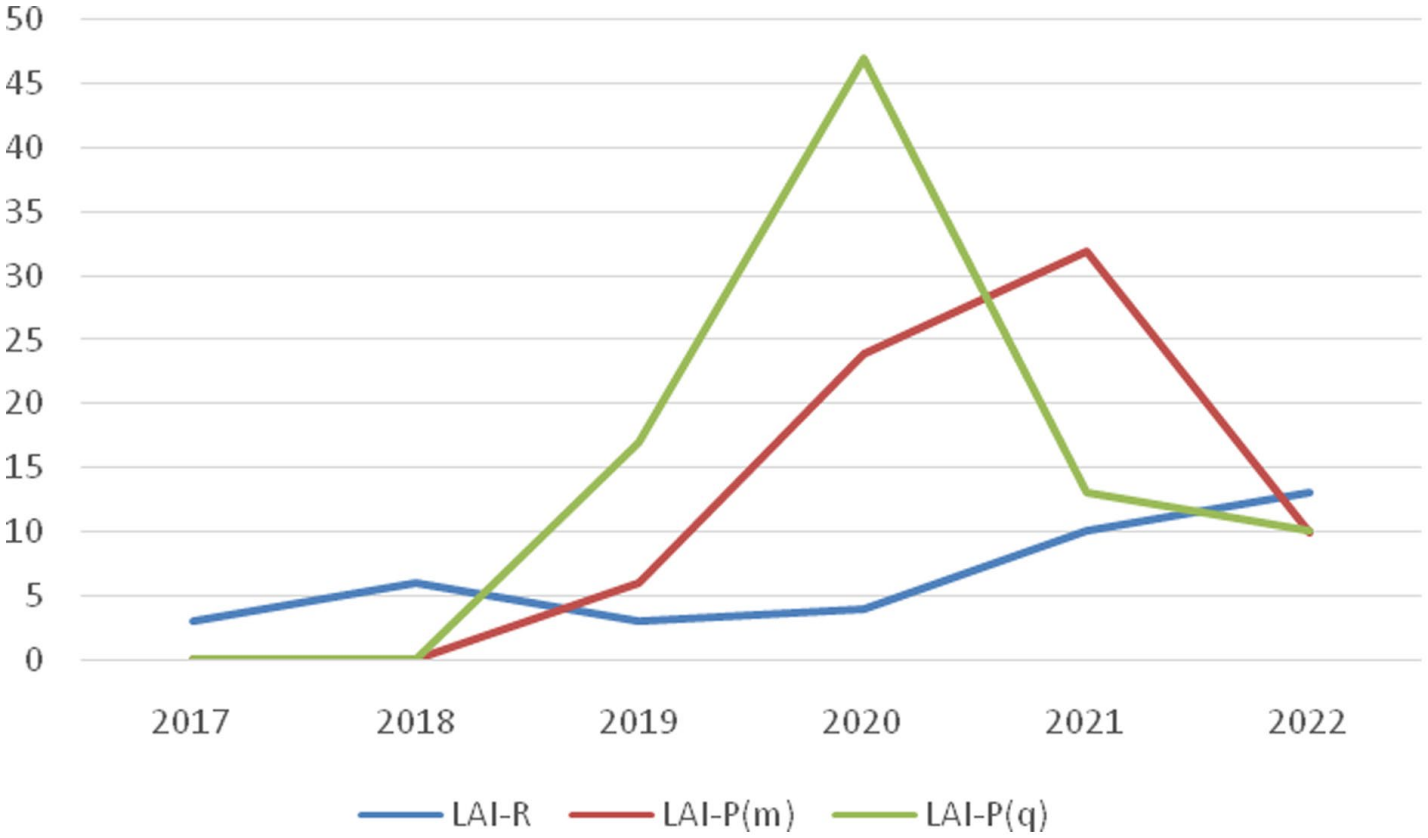
Comparison of drug prescriptions by year. LAI-R, LAI risperidone; LAI-P(m), LAI paliperidone monthly; LAI-P(q), LAI paliperidone quarterly.

Separately, the use of monthly and quarterly preparations of LAI paliperidone was significantly higher in 2020 and 2021 relative to the years before the pandemic (2017–2019). However, as the pandemic weakened, according to our research, the inclusion of LAI paliperidone therapy weakened during 2022. On the other hand, our results showed an increase in usage of LAI risperidone in 2022. There was not a significant difference in 2020 and 2021 compared to the period 2017–2019. The trend lines of usage of every followed dosage form are presented in Figure 2.

## Discussion

Our results indicated that during the COVID-19 pandemic a significant change in the application method of antipsychotic therapy favoring LAI antipsychotic therapy. LAI paliperidone had a significant increase in usage during the pandemic, while LAI risperidone was prescribed less during the pandemic. The reason for the increase in the use of medication that requires less frequent visits to health facilities should be sought in an attempt to reduce the risk of infection and consequent damage. By using these LAI, the number of patients contacts (GP, psychiatrist, nurses, pharmacist, other patients…) was significantly reduced, practically protecting them. This fact is confirmed by the data that two-week visits were on the decline in favor of monthly and quarterly visits. To our knowledge, this is the only study that compared the prescribing of LAI risperidone and LAI paliperidone before and during the coronavirus pandemic. During the literature search, we found some other studies that compared LAI of other and different antipsychotics.

### Comparison with previous studies

There are many changes that the coronavirus pandemic leads to. New techniques and methods were proposed to prevent the spreading of the virus and protect us and patients from the disease. In the literature search, there is one study that shows an increase in the use of LAI antipsychotics, they observed the use of LAI aripiprazole compared to LAI risperidone (6). A statistically significant and consistent increase in weekly aripiprazole depot mentions from March 2020 to September 2020 was observed. Risperidone depot mentions were reduced in March, June, August, and September 2020, which is consistent with our findings.

A previous study on this topic was based on creating programs that provide telehealth services to patients with schizophrenia, improving communication with patients, and promoting telepsychiatry as a new method of care during the pandemic. The onset of the COVID-19 pandemic was associated with a rapid and major shift in the mode of mental healthcare consultations. Early on, in-person visits were switched to remote (online) visits, then afterward switched back to in-person mode. They showed that the incidence of missed appointments decreased as appointments changed from in-person to remote between April and June. Telepsychiatry was suggested as a great option for many mental health problems, but schizophrenia is not one of them due to the lack of insight of these patients about their medical condition (7). During the COVID-19 pandemic, there was flourishing international scientific production, and a large percentage of published articles with data often collected through surveys or telemedicine, which have been adapted due to the impossibility of physical contact during the lockdown period (8).

### Clinical implications

LAI antipsychotics were suggested as a mainstay in the treatment of schizophrenia and related psychotic spectrum disorders because of their prolonged effect and consequent prevention of nonadherence that results in relapses and hospitalizations (9). The clinically meaningful superiority of depot medication compared to oral antipsychotics in patients with schizophrenia has been confirmed by the findings that depot formulations significantly reduced relapses from an average of 33.2 to 21.5% (10). Their usage was justified during the pandemic, even if these visits may increase the risk of infection for patients and providers (11). Although schizophrenia is considered a rare disorder, the main problem is its early onset, the chronic nature, and the course of the disease, which presents with exacerbations, frequent progressive worsening, and high comorbidity. Pharmacoeconomically the disease represents a heavy burden on society. In global consumption, direct costs related to treatment represent the lowest proportion. On the other hand, indirect costs, which are an expression of the effects of disorders and effects on care costs, occupy about 60–65% of costs (12). Adequate adherence to a treatment plan is crucial for successful mental healthcare management. For patients diagnosed with schizophrenia and another psychotic spectrum, failure to attend outpatient care following admission increases the risk of relapse and rehospitalization, which relates to larger additional medical costs. The highest costs relate to hospitalization (52.5 – 80.7% of the total costs of care). Adherence is closely related to relapses and hospitalization, as these patients will inevitably relapse at some point after drug discontinuation (13).

According to previous systematic reviews, the patient’s adherence to LAI paliperidone and LAI risperidone was slightly higher compared to oral antipsychotics, and traditional depots, although they are considerably less expensive than injectable drugs, cost the most overall for patient care due to their lower rates of effectiveness and adherence (14). Moreover, it was shown that LAI paliperidone was associated with a lower risk of healthcare resource utilization compared to oral antipsychotic therapy: in the LAI paliperidone group, the risk of an inpatient hospital admission and mental-health-related utilization was statistically lower (both *p* < 0.0001), as well as the risk of an emergency department visits (15). These results support the fact that the use of LAI antipsychotics during the COVID-19 pandemic is one of the good measures to prevent relapse. Patients who come in at two-week, monthly, or three-month intervals are at a lower risk of getting an infection, compared to patients who are on oral therapy (16).

### Pharmacoeconomic implications

There are many studies concerning the pharmacoeconomic aspects of the use of LAI antipsychotics. Compared to oral medications in a recently diagnosed group, the use of LAI paliperidone is related to higher pharmacy costs (17) Furthermore, it was shown that monthly prescription drug costs for the LAI paliperidone group were higher than the oral antipsychotic therapy group, both for all-cause pharmacy costs (*p* < 0.0001) and mental-health-related costs (*p* < 0.0001) (15).

On the other hand, a higher percent (about 55%) of the mental-health-related prescription drug cost associated with LAI paliperidone was offset by lower costs of mental-health-related inpatient and outpatient care and the (*p* < 0.0001) (15). The economic impact on the budget of prescribing LAI paliperidone appears advantaged compared to the use of oral antipsychotics, also considering second-generation antipsychotics (16). Despite a common active ingredient, treatment with LAI paliperidone represents a cost-effective choice over LAI risperidone. The therapy with LAI paliperidone has shown better treatment patterns, improvements in adherence, lower discontinuation rates, as well as a longer period of days of LAI coverage (18, 19).

Furthermore, many studies conducted across the world confirmed the advantage of LAI paliperidone’s better therapy pattern compared to LAI risperidone for treating patients with schizophrenia because of clinical and economic advantages. The lower overall cost to the healthcare system and greater clinical benefits were confirmed. The higher price of the drug was more than offset by savings accrued from less frequent drug administration and higher adherence rates. Overall, the cost of treatment has shown slightly lower for the healthcare system when PP-LAI was used, despite a higher acquisition cost (10, 20–22).

WHO reported higher health spending in response to the global COVID-19 pandemic. In 2020 reached US$ 9 trillion, or 10.8% of global gross domestic product, and was highly unequal across income groups. Higher health spending was an indicator of the priority given to health, which increased from 2019 to 2020 in all income groups except high-income countries (23).

### Limitations

This study was conducted at the University Clinical Center Kragujevac and the Faculty of Medical Sciences, and it is a local investigation. The study is limited to one center and a part of a country in Central Serbia, which may not be representative of broader prescribing patterns. The small sample of included patients, and the little ability to monitor other variables such as drug availability, patient preferences, clinician biases, comorbidities, or previous treatment history.

### Future implications

These trends can be significant in the future in the next potentially major crisis. Moreover, we can conduct surveys from the perspective of patients and their satisfaction with these prescribing trends. It is also possible to monitor the quality of life of these patients with such trends. This study can serve as an initial study for a more detailed pharmacoeconomic study and its impact on the improvement of health care.

## Conclusion

Our results showed that the use of LAI paliperidone in relation to the use of LAI risperidone increased during the lockdown period of COVID-19. One of the reasons can be that LAI paliperidone requires less frequent visits to the health clinic and therefore the patient’s exposure to risk factors is lower. Regardless of the highest pharmacy costs of LAI antipsychotics compared to oral medications, during the COVID-19 pandemic, we can assume that the safety of the patients was a priority in the treatment process. During the pandemic period, especially in years when COVID-19 restriction measures were stricter usage of therapy that allows the patients to visit the clinic less often was on the rise. In the long term, the lower overall cost to the healthcare system and greater clinical benefits were previously confirmed, and our system was able to provide more care with the same budget.

## Data availability statement

The original contributions presented in the study are included in the article/Supplementary material, further inquiries can be directed to the corresponding author.

## Ethics statement

The studies involving humans were approved by Ethics Committee of the University Clinical Center Kragujevac. The studies were conducted in accordance with the local legislation and institutional requirements. Written informed consent for participation was not required from the participants or the participants’ legal guardians/next of kin in accordance with the national legislation and institutional requirements.

## Author contributions

MiS: Conceptualization, Data curation, Methodology, Writing – original draft, Writing – review & editing. MaS: Supervision, Writing – review & editing. MJ: Methodology, Writing – review & editing. AK: Methodology, Writing – review & editing. DD: Writing – review & editing. NM: Methodology, Supervision, Writing – original draft, Writing – review & editing. MD: Writing – review & editing. SJ: Writing – review & editing. MSo: Writing – review & editing. BS: Writing – review & editing. SS: Writing – review & editing. AC: Writing – review & editing. TS: Writing – review & editing. OR: Writing – review & editing. BR: Methodology, Supervision, Writing – original draft, Writing – review & editing.
