# Supplementary material for: The impact of the COVID-19 pandemic on the trend of prescribing long-acting injections of paliperidone and risperidone in Central Serbia
Source: Front Psychiatry. 2023 Dec 21;14:1301835. doi: 10.3389/fpsyt.2023.1301835 (PMC10764607; doi:10.3389/fpsyt.2023.1301835)

Supplementary Material

Table 1: Sociodemographic data

Sex	67 females<br>130 males
Age	18 – 65 years
Dg	F20 - F29
LAI Risperidone	39 (13 females, 26 males)
LAI Paliperidone (monthly)	72 (36 females, 36 males)
LAI Paliperidone (quarterly)	87 (24 females, 63 males)

Table 2: The number of patients with LAI antipsychotics and the year

	2017	2018	2019	2020	2021	2022
LAI risperidone	3	6	3	4	10	13
LAI paliperidone monthly			6	24	32	10

LAI paliperidone quarterly	17	47	13	10

Chart 1. Prescribing of LAI before and during Covid-19

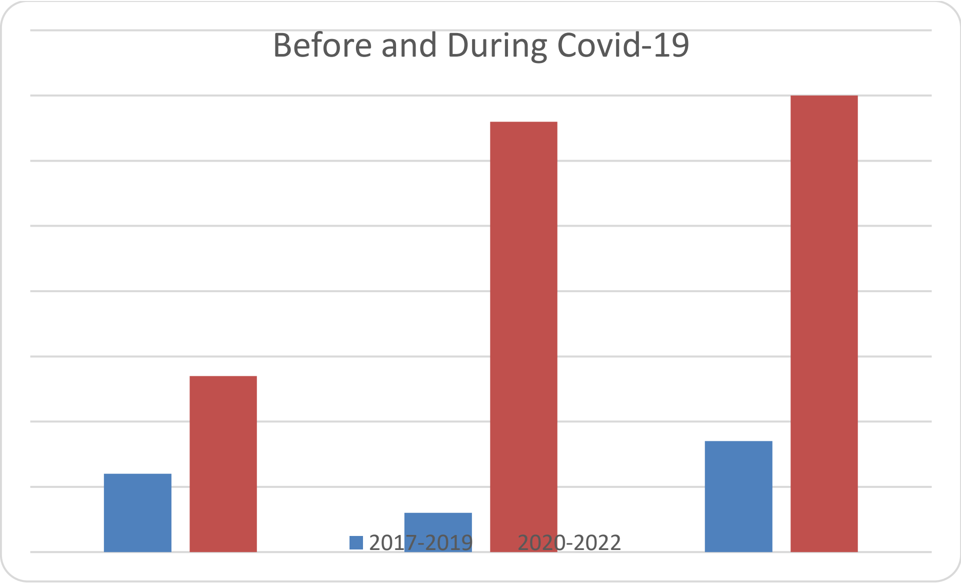

Chart 2: Individual comparation of prescribing LAI

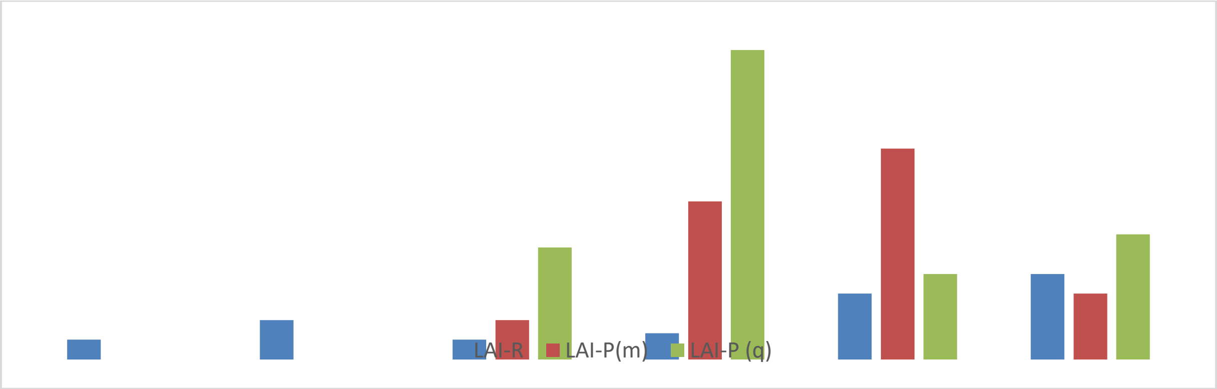

Chart 3: Comparison of drug prescriptions by year

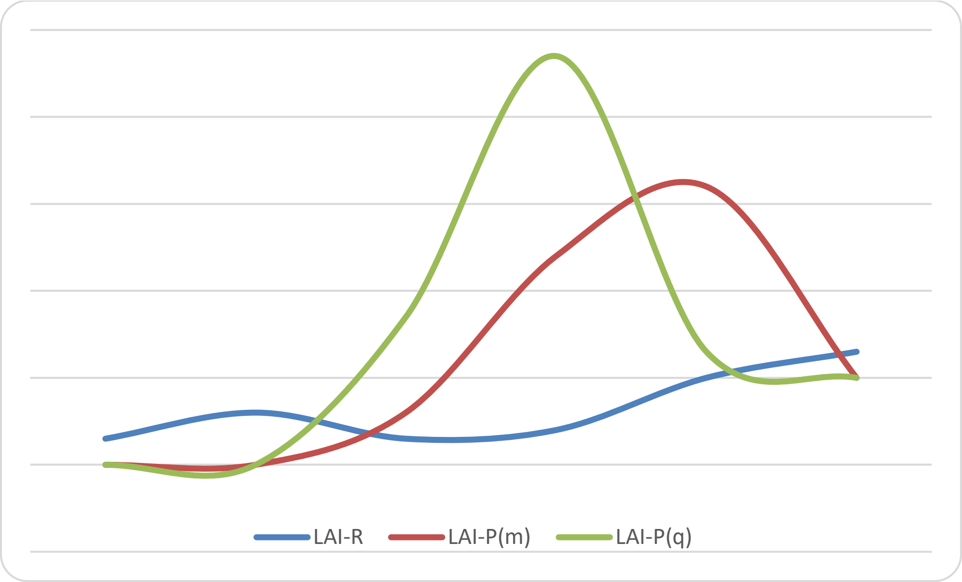

Supplement: Supplementary file 1 [file Data_Sheet_1.PDF]
